# The Repository Chemotion: Infrastructure for Sustainable Research in Chemistry**

**DOI:** 10.1002/anie.202007702

**Published:** 2020-10-07

**Authors:** Pierre Tremouilhac, Chia‐Lin Lin, Pei‐Chi Huang, Yu‐Chieh Huang, An Nguyen, Nicole Jung, Felix Bach, Robert Ulrich, Bernhard Neumair, Achim Streit, Stefan Bräse

**Affiliations:** ^1^ Institute of Biological and Chemical Systems—Functional Molecular Systems (IBCS-FMS) Karlsruhe Institute of Technology Hermann-von-Helmholtz-Platz 1 76344 Eggenstein-Leopoldshafen Germany; ^2^ Institute of Organic Chemistry (IOC) Karlsruhe Institute of Technology Fritz-Haber-Weg 6 76131 Karlsruhe Germany; ^3^ Steinbuch Centre for Computing (SCC) Karlsruhe Institute of Technology Hermann-von-Helmholtz-Platz 1 76344 Eggenstein-Leopoldshafen Germany; ^4^ KIT Library Karlsruhe Institute of Technology Straße am Forum 2 76131 Karlsruhe Germany

**Keywords:** FAIR data, open data, open science, open source, sustainability

## Abstract

The repository Chemotion provides solutions for current challenges to store research data in a feasible manner. A main advantage of Chemotion is the comprehensive functionality, offering options to collect, prepare, and reuse data with discipline‐specific methods and data‐processing tools.

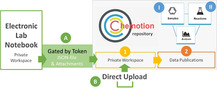

We describe the development of a repository for chemistry research data (called Chemotion) that provides solutions for current challenges to store research data in a feasible manner, allowing the conservation of domain‐specific information in a machine‐readable format. A main advantage of the repository Chemotion is the comprehensive functionality, offering options to collect, prepare and reuse data with discipline specific methods and data processing tools. For selected analytical data, automated procedures are implemented to facilitate the curation of the data. Chemotion provides functions for a feasible data publishing process including automated Digital Object Identifier (DOI) generation and workflows for peer reviewing of the submissions, including embargo settings. The described developments were used to establish a research‐data infrastructure to build a new community‐driven repository as a comprehensive alternative to commercial databases.

## Introduction

Main databases and repositories are an important component for scientific work as they allow the access to data obtained by other researchers. Therefore, databases and repositories are information systems that play a pivotal role for sustainable scientific work and they form an important basis for the dissemination and application of our current knowledge.^[1]^ An overview of the currently existing databases and repositories covering research data can be obtained by RDA (Research Data Alliance) endorsed services as DataCites re3data[^2^, ^3^] or FAIRsharing,^[4]^ as well as community specific efforts such as the Enabling FAIR Data project of the American Geophysical Union (AGU).^[5]^ These services and efforts collect resources and aggregate information from multiple sources, like the repositories themselves, publishers or persistent identifier (PID) systems. While many common chemical databases store aggregated data in form of textual representation, research data repositories are also used to store and share the underlying data in well‐structured and machine readable format, often including the raw data from scientific measurements. During the last years, an increasing number of scientists and stakeholders has realized how important research data repositories are in the view of the quality of scientific work, the reproducibility of experiments and transparency of work processes.[^6^, ^7^] Furthermore, the current progress in data analysis technologies such as machine learning, semantic technologies and big data reveals that the scientific community could benefit tremendously from large data collections which can be used for the development of intelligent information systems. Repositories could collect the data from a vast amount of mostly small size laboratories and groups of scientists that are producing most of the available data, enabling solutions for current problems related to the “long‐tail” of science.[^8^, ^9^] Altogether, research data repositories offer tools and services to address the often mentioned problems of data quality, accessibility and reusability.[^10^, ^11^] The key role that repositories play for scientific work is also recognized by publishers as many of them recommend to deposit research data in trustworthy infrastructure along with the publication itself.[^12^, ^13^, ^14^, ^15^, ^16^] The preservation and sharing of research data in repositories is not only supported by funding institutions but is also progressively enforced.[^17^, ^18^, ^19^, ^20^, ^21^, ^22^] The most often recommended general purpose repositories, FigShare,^[23]^ Zenodo,^[24]^ and Dryad^[25]^ achieved high attention during the last years. These discipline agnostic repositories offer quick solutions to data storage and access as the deposition of data can be done with only minimal preparation and costs. In any case, the relevance of these repositories with respect to the availability of machine readable and reusable data for subject specific tool sets and processing is currently limited due to the generic approach. In particular, the need for data being compliant with the FAIR (findable, accessible, interoperable, reusable) data^[26]^ principles, reveals that many of the currently available repositories lack necessary options to reuse data in a discipline‐specific manner.^[27]^

In chemistry, the discipline specific functionality of a repository is especially important, as the required information of a chemical investigation is given in a schematic presentation including structures and reactions. Processing subject specific information to provide tailored services for identification and citation, as well as collection, search and comparison, is crucial to build repositories in chemistry that enable FAIR data, and therefore, a reuse in the scientific community. So far, only a few repositories and databases in chemistry combine open access to research data and the availability of data in a reusable form. All of them are highly specific with a focus on a certain analytical method. As far as we know, the only well‐established database in chemistry that has repository functions in terms of the systematic storage of user‐provided data files, is the Crystal Structure Database (CSD) of the Cambridge Crystallographic Data Center (CCDC).[^28^, ^29^] Despite not being Open Access, most of the universities have full access to the CSD due to moderate costs for academic institutions. While most other databases or repositories in chemistry are rarely used by the scientists, the publication of crystal structures via the CSD has been accepted as a standard procedure for scientists and the repository may serve therefore as a best practice model in terms of acceptance by the research community. Only a few other very good yet rarely used examples are available for chemists, including the well‐curated databases massbank^[30]^ for mass spectrometry data and NMRshiftDB2^[31]^ for nuclear magnetic resonance shifts. Regarding synthetic information on reactions and their details for example, the purification as well as information on molecules and their analytical data, a comprehensive repository that supports the deposit and reuse of research data is currently not available. To our knowledge, the ChemSpider Synthetic Pages is so far the only initiative that provides web‐tools to deposit information on reactions and processes in chemistry.^[32]^

## Results and Discussion

**Figure 1:**
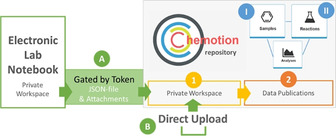
Schematic presentation of the repository architecture, structured according to reactions (I), molecules/ samples (II) and their associated analyses (blue), and the main functionality of the workspace (1) and publication area (2) of the repository (yellow and orange). Options to enter data into the repository, that is, transfer from ELN to repository (A) or upload of data from any data source (B) are marked in green.

The repository Chemotion was developed to fill the currently existing gap for the preservation and publication of experimental work in synthetic chemistry. The repository supports chemists in their efforts to store data in a reusable manner and provides functions to offer this data in a well‐structured and transparent way. The repository serves as infrastructure for scientists to deposit and share data and can be used as a source to obtain chemical data in a machine readable way. The use of the repository is free of charge for data providers and data consumers and the code of the software is available via GitHub^[33]^ as Open Source under an AGPL^[34]^ license. The repository focusses on synthetic and analytic chemistry including molecules, their properties and spectroscopic data as well as on information related to chemical reactions. With this focus, there are two main instances according to which the data in the repository are structured: samples, which represent unique batches of a molecule, and reactions (Figure 1, I and II). With this, the repository follows general management standards in chemistry where information is kept along with the experimental chemical process. Information and analytical data are assigned to either a reaction or a sample and can be correlated to those instances due to the availability of standardized identifiers such as the reaction InChI (RInChI and RInChIKey). The repository has three types of user roles: (passive) non‐registered users, (active) registered users and reviewers. Non‐registered users can access the repository and the disclosed data, but they cannot download the given information and datasets. The registered users can download any disclosed information and data files. They have access to certain functions of the repository like the workspace area, offering options for the creation of an own database and including typical, chemistry‐specific data management functions. Furthermore, only registered users are allowed to upload or transfer data to the repository. Reviewers have access to the reviewing functions of the repository that allows them to accept and decline submissions, or to ask the authors for a revision. The repository consists of the two main work areas: the workspace area (Figure 1, yellow panel) with functions to prepare, collect and manage data, and the data publication area (Figure 1, orange panel) that includes the reviewing and embargo processes. Both have a listing view, with filtering options, and a detailed view of a selected element. An important feature facilitating the use of the repository is the possibility to transfer data from an Open Source electronic lab notebook (ELN)[^35^, ^36^] into the workspace area of the repository. The process transfers all required data directly from server to server instead of having the user to manually upload each data sets and enter the metadata to the repository. Both processes, the direct transfer and the data upload are supported by the Chemotion repository (Figure 1, processes A and B).

### Workspace: Functions of the Private Database “My DB”

**Figure 2:**
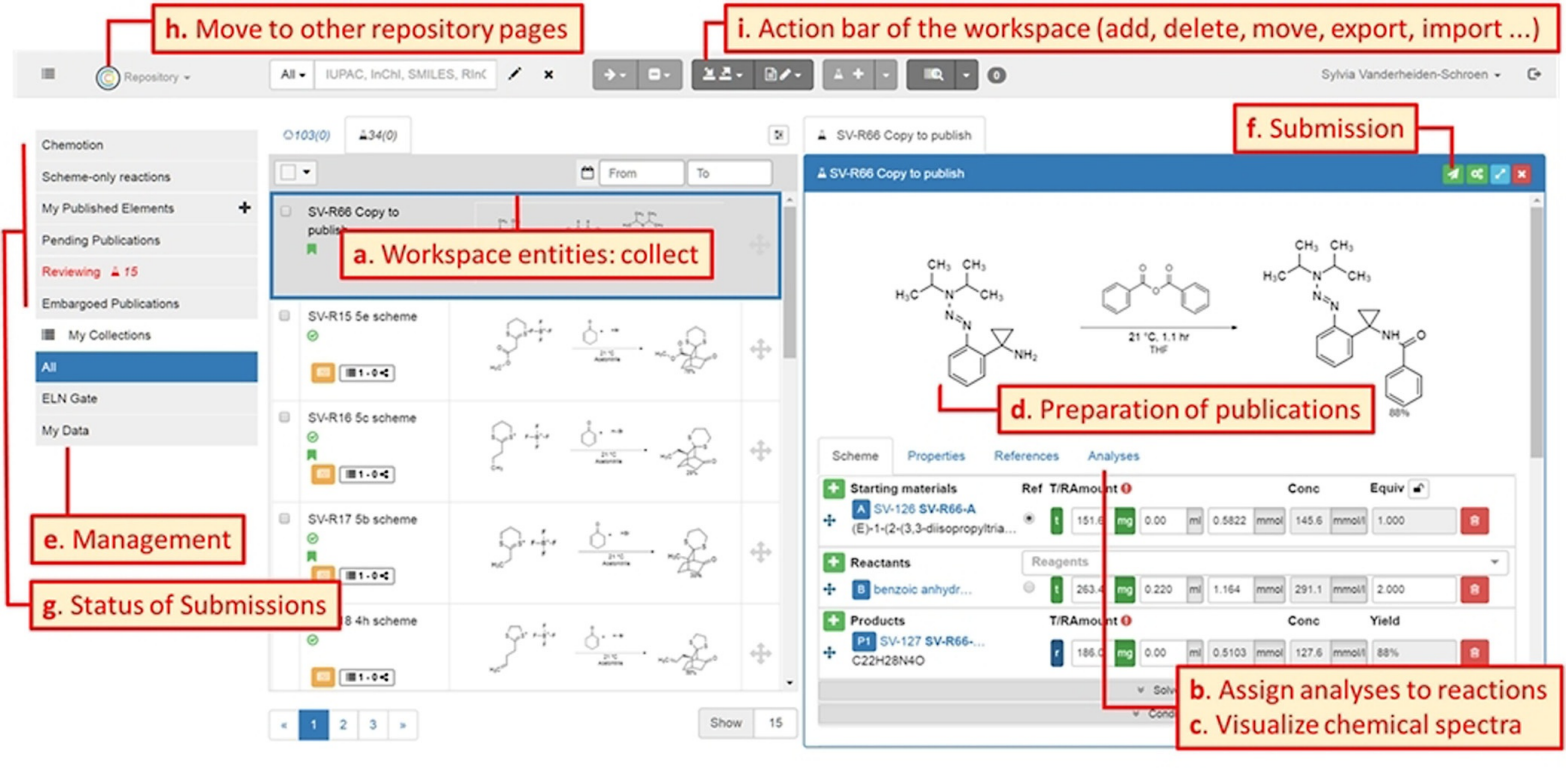
Workspace of the repository and labeling of the main functions to manage and prepare data for publication.

The workspace area is a private area only accessible to the registered user that allows to upload and manage data. It supports functions to collect, assign and visualize chemical research data (Figure 2, a–c) without the need of additional instruments or software for chemistry specific tasks. Functions such as drawing of structures with a molecule editor or automatic calculations embedded to a reaction table enable an efficient preparation of data for a publication in the repository (Figure 2, d). The discipline specific functions support the preparation of data to conserve the whole set of information for its further reuse or a detailed analysis at a later stage. This is an important advantage of Chemotion compared to generic and multidisciplinary repositories. Without options to store data according to chemistry specific standards and formats, such as the conversion of data to Word documents or PDFs, subject specific information is usually lost and the utility of the remaining data is reduced. The combination of discipline specific functions with diverse management tools (Figure 2, e) in Chemotion allows an efficient organization of textual data and analytical data files according to common documentation and reporting practices in chemistry. The workspace of the repository supports the assignment of metadata to the given datasets by dropdown menus or text fields, depending on the type of information. Users are requested via notification to provide the necessary metadata before submitting to the data publication area. The metadata fields available as dropdown support the vocabularies of the Chemical Reactions Ontology (RXNO)^[37]^ and Chemical Methods Ontology (CHMO).^[38]^ Beside the preparation of the data for submissions, the workspace area of the repository is also the place where the publication process can be started (Figure 2, f), or ongoing processes, such as the review, can be followed (Figure 2, g and h). Using the workspace is also helpful for registered users who may benefit from functions like the export of data or the visualization of data with a chemistry specific data viewer. In particular, the export options (Figure 2, i) are important to access machine readable data. To the current state, the workspace supports the export of selected information to MS‐Excel, and Word document, or SDF,^[39]^ but also the extraction of collections as zipped data containing a JSON file and the assigned experimental data files. Discipline specific as well as management functions of the workspace in the repository are summarized in Table S1 (Supplemental Information).

The repository Chemotion offers the option to transfer data from an Open Source ELN to the repository workspace with only a few clicks. This function was designed from the experience made by other initiatives to collect research data. The success of a repository or database and its acceptance in the community depends strongly on the ease of use regarding upload and curation of the data to be deposited.[^40^, ^41^] Therefore, the use of a repository should be as simple, automated and user friendly as possible for the data providers. Unfortunately, the effort for repository submissions increases with the complexity and diversity of the data. The effort increases even more with the needs for sustainability and reuse since those require for example, completing metadata or organizing the data differently to support varying use cases such as machine‐to‐machine communication or cross‐disciplinary reusability. The most feasible option to provide sustainable data is to make use of already well‐organized, curated data and their interoperable transfer to a repository. The data transfer routine from an ELN to a repository demonstrates the effortless and barrier‐free transfer of data from a work environment of scientists and forms a key asset to encourage scientists to deposit their data in Chemotion. The interoperable data transfer is currently realized for the Open Source Chemotion ELN that serves as an example for connecting other ELNs in future (please see Supplemental Information for a description of the application programming interface (API) and methods to be implemented). The data transfer is realized via a special work area within the ELN allowing the user to copy and transfer the data. After activating the personal repository account and linking it to the users’ ELN account, the data can be mirrored with two additional clicks to initialize and confirm the transfer. The technical transfer includes a JSON file format and associated data files (see methods section).

### Publication Process

**Figure 3:**
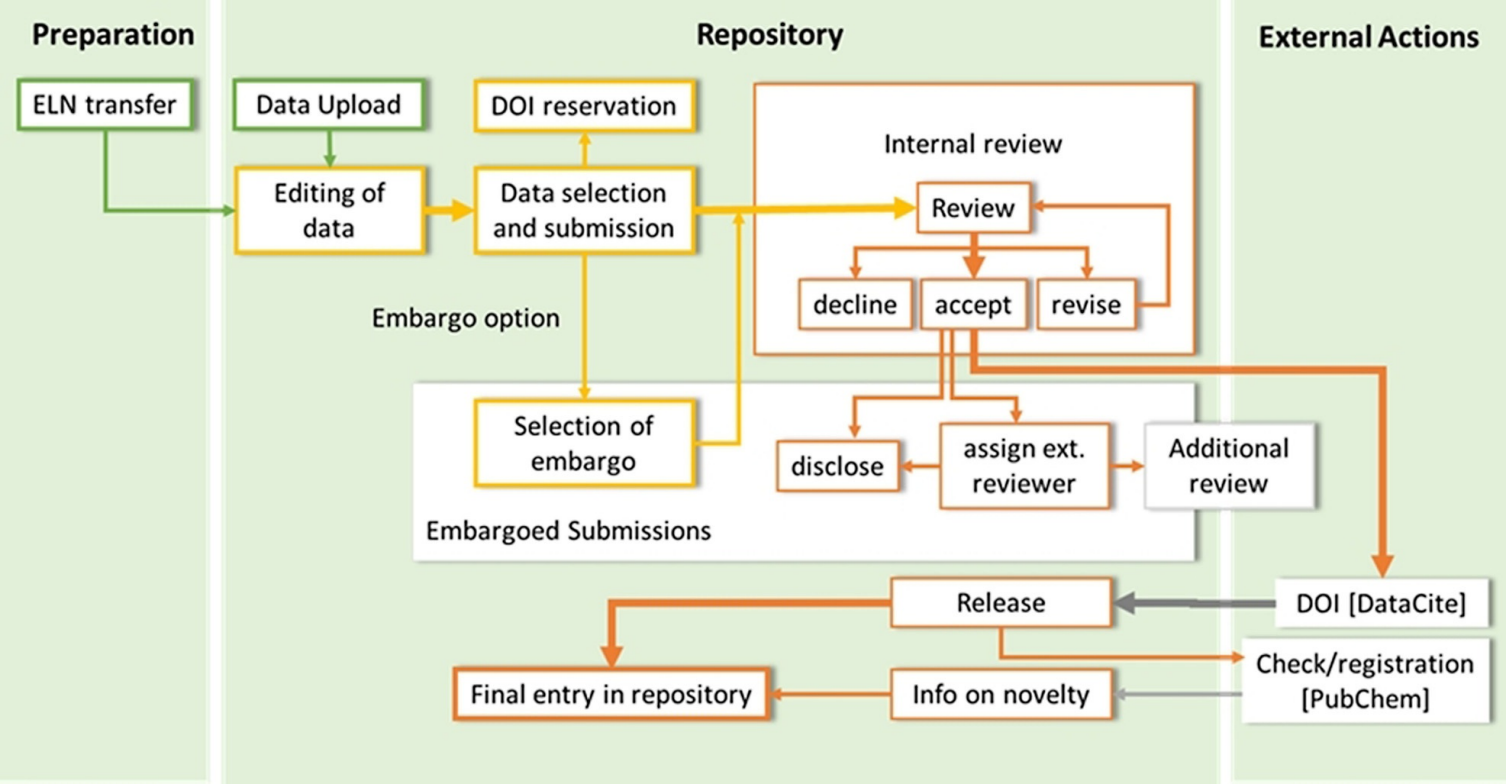
Workflow including optional transfer of data from ELN, the submission with and without embargo, and the reviewing process including mandatory review by the repository team or optional review by additional external reviewers. The single stages towards the final publication entry in the repository are assigned to three main parts: the preparation of the data, workflow of the repository and participation of PubChem and DataCite. Color coding refers to Figure 1. green=data provision, yellow=repository workspace area (1); orange=repository publication area (2). The fastest workflow is highlighted by bold arrows.

**Figure 4:**
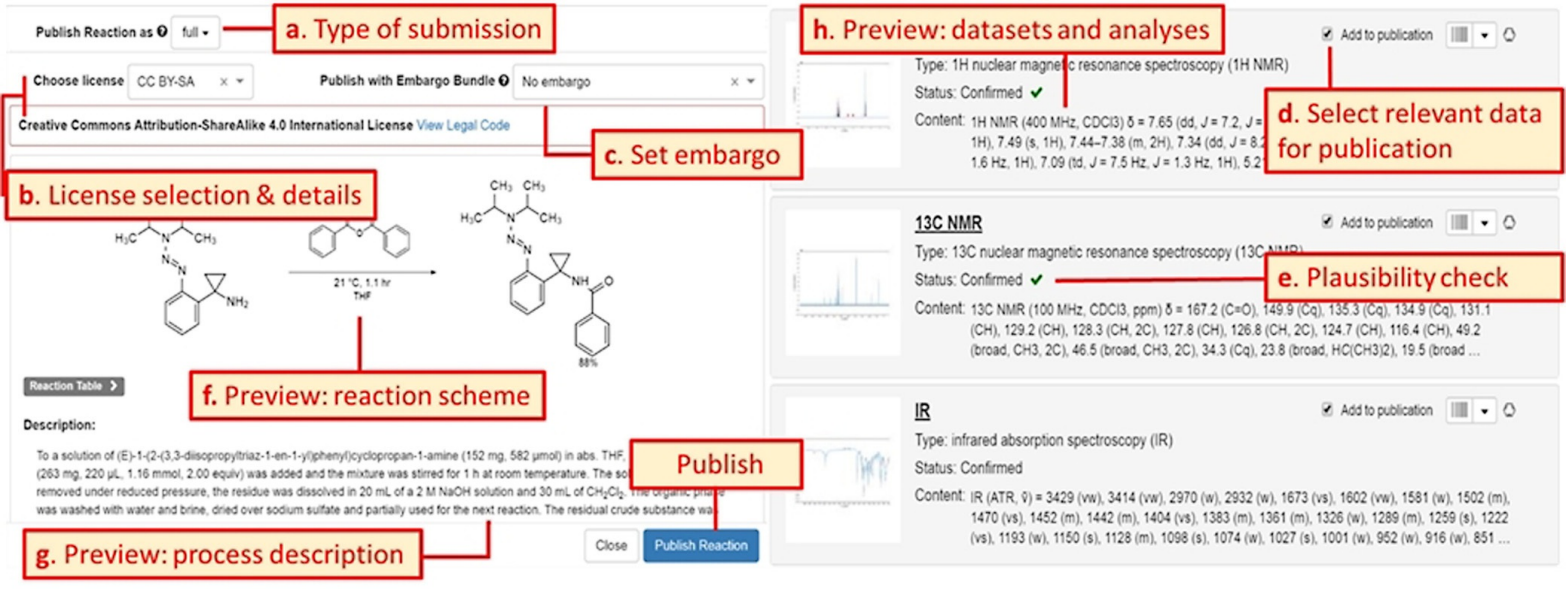
Publication panel to be edited and checked by the data provider. The publication panel is part of the submission process within the workspace of the repository (Figure 3).

The scientists who decide to publish their data can start the publication process via uploading data to the repository or using the interoperable transfer of data from an ELN instance (green colored panels, Figure 3). In both cases, data can be managed and edited in the repository workspace until the latest version is marked as finalized. Within the workspace (yellow colored panels, Figure 3), datasets can be selected to submit them along with a reaction or a molecule/ sample (detailed description in Figure 4).

All data can be submitted either with or without an embargo and are subjected to the internal reviewing that focuses on plausibility and formal issues of the submission. The internal review can result in a request to revise the submission. After improving and resubmitting the data, the reviewers can either decline, accept or send back the submission to the author for another review. For accepted submissions, the repository mints a Digital Object Identifier (DOI) automatically, so the dataset can be cited and referenced. No user interaction is necessary. For embargoed submissions, the release of the dataset is on hold until the owner decides to end the embargo and disclose the data. While the data are under embargo, additional external reviewers with read level can be assigned, allowing the review of the data while the publication process is on hold (Figure 3). The external review supports anonymous access to the data if a publication is submitted to a peer reviewed journal where the peer reviewers should be able to review also the data belonging to the manuscript. In parallel to the release of the data via the Chemotion publication site, the DOIs are generated. The released molecule structures are checked for their presence in the database PubChem to give information on the novelty of the compounds. Yet known and unknown molecules are registered with their Chemotion reference in PubChem as “PubChem substance”. The submission of the data comprises the cloning of the data from the private workspace to the publication area of the repository. This transfer is managed by a publishing panel that is available per molecule or reaction submission process (Figure 4). For each bundle of data, the user is requested to select and fill necessary information such as the submission type, licenses, embargo(‐bundles),^[42]^ relevant data files (Figure 4, a–d) and co‐authors (not shown in Figure 4) allowing the user‐defined disclosure of the data. The publication panel gives additional information on the plausibility of the given data^[43]^ (Figure 4, e), and supports to check the submission by previewing the metadata, the process descriptions as well as datasets and analyses (Figure 4, f–h). The repository Chemotion supports the generation of DOIs as a de facto standard persistent identifier, which is provided by DataCite. The name of the DOI is defined by rules of the repository (e.g. including InChIKey^[44]^ of the molecules, reactions and their versions), and can be reserved prior to the DOI minting. This enables the user to preview the DOI of a dataset before it is definitively registered with DataCite during the formal release of the data. This allows the authors to use the DOI and cite a dataset in the Supplemental Information of their text publications even before the data are visible to the public, thus providing a beneficial function to prepare manuscripts. The review functions of the repository are only accessible to reviewers and authors. Reviewers access the reviewing area through a landing page that summarizes the current requests and reviewing processes (Figure S1, Supplemental Information). Authors have access to the review functions via the workspace. The reviewer can comment on each information that was provided by the author allowing a detailed response on the provided data and their analysis. As soon as the review is completed, the author receives a notification about the review result, which may include a link to the data to be corrected. The user has also the option to reply to the reviewers’ requests and comments. The procedure of reviewing, correction and resubmission can be repeated as often as necessary and desired, but reviewers may also reject a submission if the data or the manner of correction does not meet the desired quality standards. In the long term, the peer reviewing process should be supported by automated tools to check data prior to a peer review. The repository is an excellent basis to develop such automated curation and reviewing tools as the information on chemical structures that is calculated and predicted can be compared to experimental results or the manually given analysis. Such a plausibility check is demonstrated by an implementation that supports the quick evaluation of NMR data: the repository compares the theoretically given numbers of hydrogens and carbons (accessible via the formula) with the practically found ones (analysis of ^1^H NMR and ^13^C NMR data) and indicates whether the NMR data given are plausible or not. This example demonstrates how simple tools may improve the quality and facilitate the curation of data or support the peer review process.

### Disclosed Information and Data

**Figure 5:**
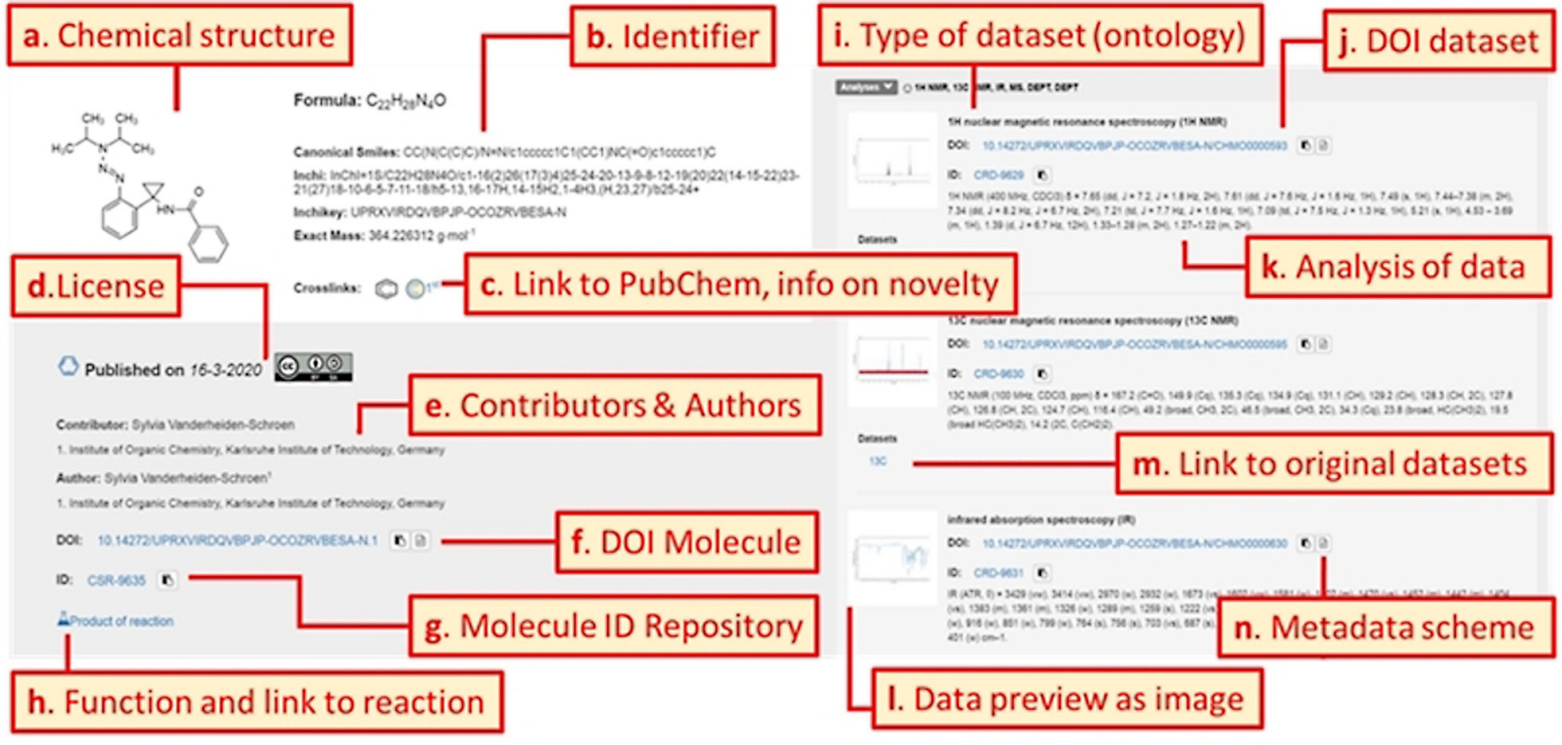
Landing page for an exemplary molecule publication in the repository and labeling of the most important information and links.

The publication area of the repository is accessible without any restrictions or the need to register, therefore the data are directly visible to the public after its disclosure. According to the structure of the workspace, (analytical) data are assigned either to a reaction or a molecule also in the publication area. Native relations like the affiliation of molecules to their function as starting material or product of a reaction are conserved in all processes of the repository. Vice versa, published reactions always contain the correlation and direct links to the assigned molecules and their data. Figure 5 shows an example for a landing page of a molecule and the data that are assigned to this molecule. An example for the landing page of a reaction is given in the Supplemental Information (Figure S2). The publication landing page of reactions contains similar information in terms of the provided general metadata but it differs in the reaction specific part that consists of the description of chemical processes. Based on the given information, that is, the molecular structure in the depicted example (Figure 5 a), the repository provides additional and automatically generated data to improve the machine readability, searchability and citation of the data. For molecules, the publication landing pages contain metadata describing the molecule with the most important molecule identifiers (SMILES, InChI, InChIKey), the IUPAC name or formula^[45]^ of the molecule and its exact mass (Figure 5 b). In case of a reaction, the information provided by automated processes of the repository consists of the standardized reaction identifiers RInChI, RInChIKey, Short‐RInChIKey and Web‐RInChIKey (Figure S2). If the submitted molecule is already known in PubChem,^[46]^ which is used as the reference database for already described molecules, a crosslink gives the direct connection to the entries in PubChem. New molecules are indicated by the information symbol “Chemotion First”, showing that the molecule was not known at the time of the submission to the repository (Figure 5 c). Every submission is given with metadata on the submission date, involved contributors, authors, and their affiliations as well as the license that was selected during the submission (Figure 5, d and e). The implementation of the OAI‐PMH also makes this metadata of the published repository entries available to harvester systems. The DOI, including a versioning indicator^[47]^ for different submissions for one molecule or reaction, is given along with a unique reference number, the repository entry ID, which is registered for all published molecules, reactions and their data (Figure 5, f, g and j). Every product of a chemical reaction, for which the process is described in the repository, is linked to the corresponding data (Figure 5, h). Literature references can be assigned to the molecules and reactions by the author or by other registered users at any time (see Figure S2). The data assigned to the molecule consist typically of analytical information from different measurements which are sorted according to the single available sample submissions (Figure 5, right panel). The single datasets, for example, from analytical processes, consist of additional metadata such as the type of the dataset defined by an embedded ontology (Figure 5, i), the DOIs of each dataset (Figure 5, j), and the analysis/interpretation of the data including the corresponding deposited file types (Figure 5, k). Data previews and the analysis of the data give embedded insights about the content of the datasets. All related data files are accessible via the landing page and can be downloaded by registered users (Figure 5, l and m). The data listed for a reaction, in comparison to the data for a molecule, contain the structure information of the reaction, the reaction table including all reagents and solvents, the conditions of the reaction, and experimental details on the purification of the reaction mixture. Molecules that are assigned as products of the reaction and their analytical data are also referenced and visualized with the full information (Figure S2). For both instances, molecules and reactions, metadata submitted to DataCite matching their metadata scheme, can be downloaded (Figure 5, n) (an example is added to the SI).

The repository Chemotion offers a platform to preserve and disclose scientific data in the domain of chemistry with a strong focus on synthetic chemistry and analytical chemistry. Such a research data repository for reactions including analytical data was missing worldwide so far. The major aims are to establish progressively a large database that supports the needs of chemists in chemical synthesis and that meets the growing need of machine readable and FAIR data. Several milestones to reduce the technical hurdles for scientists to provide data have been reached with Chemotion and its underlying software. The challenge of collecting a large number of datasets can be solved in the long‐term by hosting the Chemotion repository for the research community at Karlsruhe Institute of Technology (KIT).^[48]^ Our approach shows that the combination of an ELN and a repository can be used to transfer data, in a barrier‐free and effortless manner, from a work environment to an Open Access repository. This level of interoperability between an ELN and a repository regarding data transfer is, as far as we know, unique at least in the scientific domain of chemistry. The data deposited is machine readable wherever possible and several functions including the generation of identifiers, the link to external databases as well as export and reporting functions allow the comprehensive reuse of the data also for humans. However, there are some limitations with respect to machine readability and standardization that the repository cannot solve at the moment: The repository allows the description of processes in common formats and the disclosure of all types of data files. For certain types of data, a mechanism was established that reminds the user to provide a digital file format which is requested in the reviewing process. It is strongly recommended to store data in open file formats such as JCAMP^[49]^ for spectroscopic data in general, or NMReData^[50]^ for NMR in particular. The recommendation of these file formats is given to the user through instant notifications. Nevertheless, as probably for many other disciplines, there are currently only a few mandatory standards for the description of (chemical) processes and (analytical) datasets so that the repository can only implement procedures for established standards. Hence, the further development of the repository in terms of the supported mandatory standards depends strongly on the chemical community and other stakeholders. Recommendations known to the current date are used to establish a best practice procedure for the deposition of data in chemistry and we aim to contribute with those suggestions to suitable models for future standardization. For now, the internal reviewing team decides on submissions that do not meet the best practice model depending on the overall quality and reusability of the data. These review activities, along the recommendations to best practices and standards, will be extended continuously and adapted to the outcome of international initiatives[^51^, ^52^] and projects.^[53]^

To foster the acceptance of the repository by data providers, the repository was set up with a rich functionality to serve data providers with manifold functions such as the extensive workspace area, the reviewing function, and embargo settings. The main repository instance at the KIT in Karlsruhe is open for any contributions of the community as long as the single dataset or contributions of single users meet the desired quality criteria and the size of the data files can be reasonably justified. This allows the establishment of a large, reusable database of reactions and data on molecules created by the scientists. The Open Source code of the repository available at GitHub allows scientists to host a repository at their institute as a local archiving system and to benefit from the developed system even if it is not desired to disclose data to the public directly. Such a procedure will prevent the loss of data and will allow to merge data with the repository hosted in Karlsruhe at a later stage if desired.

The repository as presented here will be extended with several functions, such as a procedure to add information on reactions without attached analytical data, in future. A fast process for the extraction of literature‐known information in combination with a registration in the repository will allow a fast enrichment of the database with additional information on established reactions. Forthcoming developments will also include the extension of the currently supported metadata information in the DataCite metadata scheme.

## Conclusion

Currently, the chemistry community lacks domain specific open access repositories and databases that serve the needs of scientists with respect to data storage and search for data, their reuse as well as data analysis. While the expectations of funding agencies and publishers in terms of data management and the storage and disclosure of research data increase continuously, the scientific community faces problems to meet these requirements due to a missing or yet insufficient research data infrastructure. We describe the development of a repository for chemistry research data (called Chemotion) that provides solutions for current challenges to store research data in a feasible manner, allowing the conservation of domain specific information in a machine readable format. A main advantage of the repository Chemotion is the comprehensive functionality, which offers options to collect, prepare and reuse data using discipline specific methods and data processing tools. For selected analytical data, automated procedures are implemented to facilitate the curation of the data. Chemotion provides functions to facilitate the publishing process of data and the citation of the deposited data. It supports automated Digital Object Identifier (DOI) generation, the comparison of the submissions with PubChem instances, and workflows for peer reviewing of the submissions including embargo settings. The described developments were used to establish a research data infrastructure that is hosted at the Karlsruhe Institute of Technology (KIT), including the necessary storage and support to build a new community‐driven repository as a comprehensive alternative to commercial databases. The use of the repository is free of charge and the data are provided and licensed as Open Data. In addition, the software is available at GitHub as Open Source and the code can be used by all interested scientists or institutions, to operate their own repositories with chemistry specific functions that enable sustainable data storage.

## Conflict of interest

The authors declare no conflict of interest.

## Supporting information

As a service to our authors and readers, this journal provides supporting information supplied by the authors. Such materials are peer reviewed and may be re‐organized for online delivery, but are not copy‐edited or typeset. Technical support issues arising from supporting information (other than missing files) should be addressed to the authors.

Supplementary
