# Supplementary material for: The Repository Chemotion: Infrastructure for Sustainable Research in Chemistry
Source: Angew Chem Int Ed Engl. 2020 Oct 7;59(50):22771–8. doi: 10.1002/anie.202007702 (PMC7756630; doi:10.1002/anie.202007702)
Supplement: Supplementary file 1 — Supplementary [file ANIE-59-22771-s001.pdf]

Angewandte Chemie
Eine Zeitschrift der Gesellschaft Deutscher Chemiker

Supporting Information

The Repository Chemotion: Infrastructure for Sustainable Research in Chemistry**

Pierre Tremouilhac, Chia-Lin Lin, Pei-Chi Huang, Yu-Chieh Huang, An Nguyen, Nicole Jung, Felix Bach, Robert Ulrich, Bernhard Neumair, Achim Streit, and Stefan Bräse**

anie_202007702_sm_miscellaneous_information.pdf

Supplemental Information

Contents

1. Availability and requirements 2
2. Summary of the generic management as well as domain specific functions of the workspace area... 2
3. Methods 3
4. Further figures describing the functions of the repository 4
5. Metadata schemes generated with Chemotion repository 5
6. Description of the API and methods to transfer data to the repository 7
7. Information on entities that occur in more than one version 8
8. Further details: IUPAC name versus formula 9

1. Availability and requirements

Project homepage for ELN and repository: eln.chemotion.net
Web access to the repository installation at KIT: https://www.chemotion-repository.net/welcome
Videos and explanations on youtube channel of chemotion (ELN and repository): https://www.youtube.com/channel/UCWBwk4ZSXwmDzFo_ZieBcAw
Operating system(s): platform independent access, developed/tested on Linux and Mac, deployed on Linux.
Other requirements for users: Modern internet browser supporting HTML5 and JavaScript.
Recommended browsers: Chrome
Programming language: JavaScript, Ruby, Python
Source Code on Github (ELN): https://github.com/ComPlat/chemotion_ELN
Source Code on Github (repository): https://github.com/ComPlat/chemotion_REPO
Zenodo link to the Source Code: https://doi.org/10.5281/zenodo.3755769
Using the virtual machine template for ELN: the virtual machine templates and explanations how to use them can be accessed here: https://git.scc.kit.edu/ComPlat/chemotion_eln_server/-/wikis/vm-templates/
To import the template, a VirtualBox from Oracle can be used: https://docs.oracle.com/cd/E26217_01/E26796/html/qs-import-vm.html
Articles on how to configure the VM network: https://www.nakivo.com/blog/virtualbox-network-setting-guide/ or https://www.virtualbox.org/manual/ch06.html#natforward.
License: AGPLv3

2. Summary of the generic management as well as domain specific functions of the workspace area

Table S1. Most important functions that are accessible *via* the workspace area ("My DB")

Functions	Details
Search	Advanced text search: Search for labels, (chemical) identifiers
	Structure search: substructure and similarity search
Management	Management of elements according to collections and processes
	Predefined areas for data at several stages of reviewing process
	Management of own entries and published data from others in list format
	Summary of key indicators in list summaries (starting materials, products)
	Management of lists by search filters or date
	Overview of submission status of own submissions
	Export samples or reactions
	Information on PubChem existence and link for all molecules
Export	Export single molecules or reactions or groups of both to Excel
	Export collections including data files (currently limited to own submissions)
	Export of molecules to SDF
	Export of reactions to different Reaction InChI(Key) formats
	Export of information to Word

References	Add references to new or existing entries
Visibility: Details	Molecular mass and exact mass for molecules
	Names and/ or formula of molecules
	Links
	Properties and further information on samples if available (e.g. purity, density)
	Further details on the reaction details such as purification details
Analyses	Download of analyses for samples and reactions (if available)
	Further information on the instruments used and description of methods
	Further description of the analytical content
Authors functions:	Collect and sort analytical data and assign analytical data
Data preparation	Collect “status unconfirmed” and approve “status confirmed” data
	Check NMR analysis by quick check procedures
	Attach data files and give details to the methods and procedures
	Process analytical data and generate previews
	Select methods from CHMO-ontologies
Review Process	Start submission an review process
	See referees’ comments and change data
	Resubmit data

3. Methods

The Chemotion Repository software is built based on the software Chemotion ELN. The software is a web application having the back-end server built on the Ruby on Rails framework[1] with PostgreSQL relational database, and the front-end user interface is mainly constructed with the ReactJS framework[2] to serve single page applications. The repository code is regularly updated with the latest developments of the Chemotion projects on GitHub by git rebasing. Features that are non-essential to the repository functions are simply disabled and hidden from the end-user. The current code is available through Zenodo.[3] The data from a Chemotion ELN instance is transferred to the repository using https, with the data serialized as a JSON object, while the analyses files are uploaded as MIME multipart attachments. The transfer request is authorized and authenticated using a JSON Web Token that the end-user previously fetched from its repository account and registered with its ELN account. The IUPAC identifiers that are available through the repository for each entry are generated using an Openbabel implementation of the InChI software (v1.05). RInChI is generated using Rinchi-gem[4] a ruby binding gem of the InChI core software (v1.05) and the reaction InChI/ RInChI (v.1.00).[5] To register the publication DOI metadata with DataCite *via* the Metadata Store (MDS) API, the Metadata Schema Version 4.3. DataCite e.V.[6] is used. The metadata of the publication can be accessed by machines either by using DataCite DOI services or directly from the repository service using the protocol for metadata harvesting from the Open Archive Initiative (OAI-PMH).[7] For a registration of samples in the database PubChem, publication samples are registered as PubChem substances using the PubChem upload services.[8,9] Technically, molecule structures are sent as molfiles *via* ftp. The molfiles are supplemented with a unique and repository specific identifier. Upon review and acceptance from PubChem they are assigned a PubChem substance ID (SID). Background jobs in the repository regularly query the PubChem substance and compound databank through the PubChem REST API to fetch the correspondingly assigned SID for the sample and compound ID (CID) for the molecule.

4. Further figures describing the functions of the repository

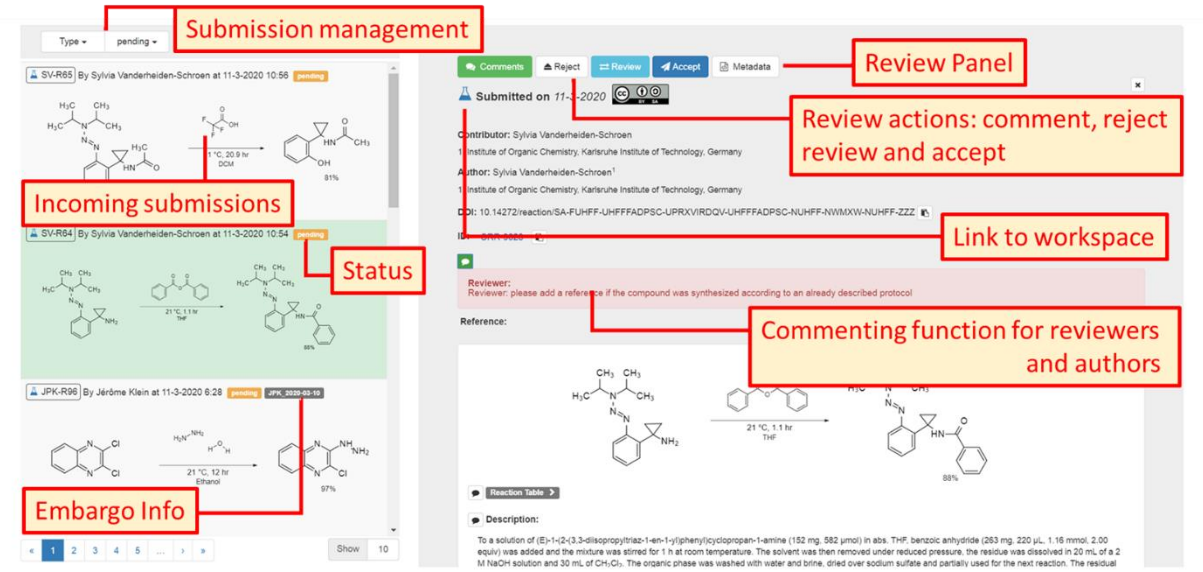

Figure S1. View of a typical review panel that summarizes the submitted data for the reviewers. The data can be checked and commented directly or the reviewer can access the workspace for a detailed view and data check.

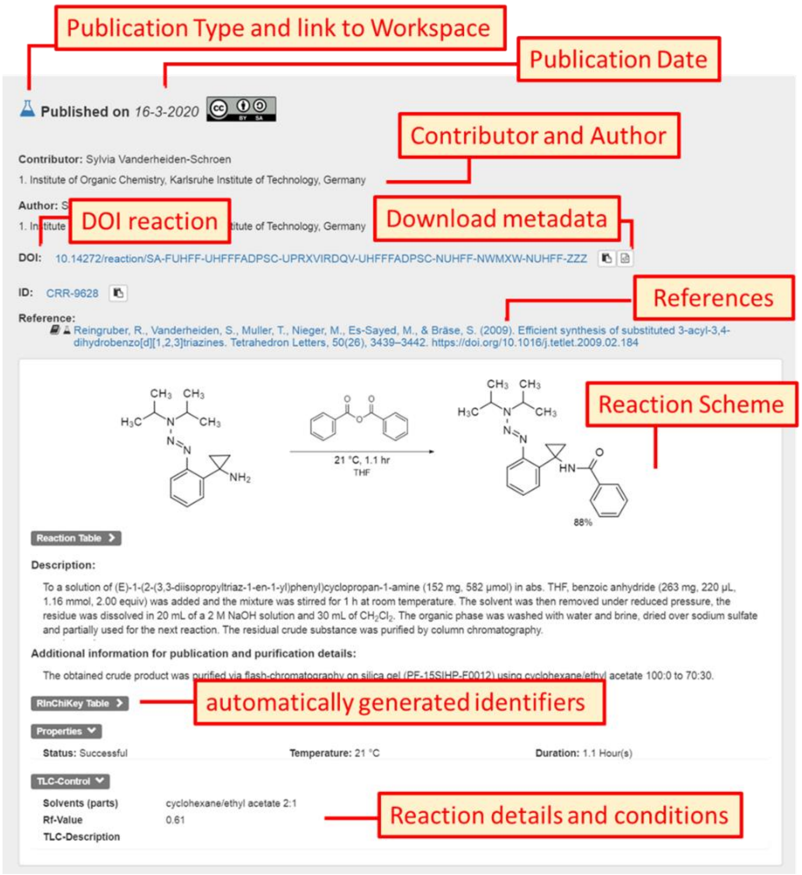

Figure S2. Landing page for an exemplarily selected reaction publication in the repository and labeling of the most important information and links.

5. Metadata schemes generated with Chemotion repository

Example of Metadata for a reaction registered at DataCite:


```
<?xml version="1.0" encoding="UTF-8"?>
<resource xmlns:xsi="http://www.w3.org/2001/XMLSchema-instance"
xmlns="http://datacite.org/schema/kernel-4"
xsi:schemaLocation="http://datacite.org/schema/kernel-4
http://schema.datacite.org/meta/kernel-4.3/metadata.xsd">
  <identifier identifierType="DOI">10.14272/reaction/SA-FUHFF-UHFFFADPSC-
FWQMCAAAAI-UHFFFADPSC-NUHFF-NBSLK-NUHFF-ZZZ</identifier>
  <creators>
    <creator>
      <creatorName nameType="Personal">Gräßle, Simone</creatorName>
      <givenName>Simone</givenName>
      <familyName>Gräßle</familyName>
      <affiliation>Institute of Organic Chemistry, Karlsruhe Institute of
Technology, Germany</affiliation>
      <affiliation>Institute of Biological and Chemical Systems - Functional
Molecular Systems, Karlsruhe Institute of Technology, Germany</affiliation>
    </creator>
  </creators>
  <titles>
    <title xml:lang="en-US">Short-RInChIKey=SA-FUHFF-UHFFFADPSC-FWQMCAAAAI-
UHFFFADPSC-NUHFF-NBSLK-NUHFF-ZZZ</title>
    <title titleType="AlternativeTitle">Short-RInChIKey=SA-BUHFF-FWQMCAAAAI-
AXXMBEHRGZ-XTFCWHIVPN-NBSLK-NUHFF-NUHFF-ZZZ</title>
  </titles>
  <publisher>chemotion.net</publisher>
  <publicationYear>2020</publicationYear>
  <contributors>
    <contributor contributorType="Researcher">
      <contributorName>Gräßle, Simone</contributorName>
      <givenName>Simone</givenName>
      <familyName>Gräßle</familyName>
      <affiliation>Institute of Organic Chemistry, Karlsruhe Institute of
Technology, Germany</affiliation>
      <affiliation>Institute of Biological and Chemical Systems - Functional
Molecular Systems, Karlsruhe Institute of Technology, Germany</affiliation>
    </contributor>
  </contributors>
  <dates>
  <date dateType="Created">2020-04-06 08:40:09 UTC</date>
  </dates>
  <subjects>
    <subject>chemical reaction: structures conditions</subject>
  </subjects>
  <language>en</language>
```

```
  <resourceType resourceTypeGeneral="Workflow">reaction conditions</resourceType>
  <version>1</version>
  <rightsList>
    <rights xml:lang="en-US" schemeURI="https://spdx.org/licenses/"
rightsIdentifierScheme="SPDX" rightsIdentifier="CC-BY-SA-4.0"
rightsURI="http://creativecommons.org/licenses/by-sa/4.0/">Attribution-ShareAlike
4.0 International (CC BY-SA 4.0)</rights>
  </rightsList>
  <descriptions>
    <description xml:lang="en-US" descriptionType="Abstract">
      A solution of N-propan-2-ylpropan-2-amine (1.03 g, 1.33 mL, 10.2 mmol, 1.10
equiv) in a mixture of 27 mL of dry THF and 3 mL of pyridine (9:1) was slowly added
to a solution of 2,4-dibromo-6-cyanobenzenediazonium tetrafluoroborate (3.46 g,
9.23 mmol, 1.00 equiv) in 10 mL of acetonitrile at -20 °C under nitrogen
atmosphere. The cooling was removed and the mixture was stirred at 21 °C for 18
hours. For work-up water was added and the mixture was extracted with methylene
chloride. The combined organic layers were dry with Na2SO4, filtered and the
solvent was removed under pressure.
    </description>
  </descriptions>
  <relatedIdentifiers>
      <relatedIdentifier relatedIdentifierType="DOI"
relationType="HasPart">10.14272/FWQMCAAAAITRGC-ISLYRVAYSA-N.2</relatedIdentifier>
  </relatedIdentifiers>
</resource>
```


The generated metadata are either retrievable directly by the user for a specific entry on the repository publication page or by machines by harvesting from DataCite services, or by harvesting using the OAI-PMH protocol at http://www.chemotion-repository.net/oai-pmh
(eg: http://www.chemotion-repository.net/oai-pmh?verb=GetRecord&metadataPrefix=oai_dc&identifier=10.14272/reaction/SA-FUHFF-UHFFFADPSC-ALWWYBOVTK-UHFFFADPSC-NUHFF-NUHFF-NUHFF-ZZZ)

6. Description of the API and methods to transfer data to the repository

A user can transfer programmatically sample data to its Chemotion repository account. For this, one needs to retrieve an access token by visiting: https://www.chemotion-repository.net/pages/tokens

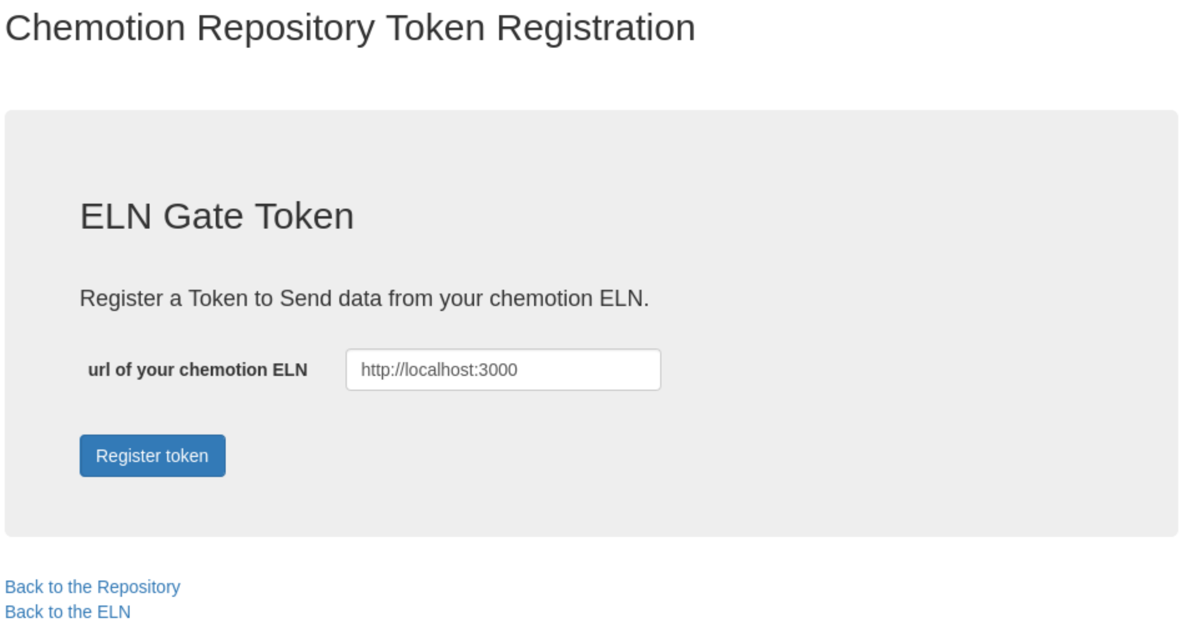

Figure S3. Adding the required URL to generate the token at the repository.

In place of the URL field, one has to enter the origin from where the transfer request will be run. The access token can then be extracted from the url of the redirected page.

For an example data can be transferred using a curl command:
curl -H 'Accept: application/json' -H 'Authorization: Bearer <user token>' -H 'origin: http://localhost:3000' -F data=@data.json https://www.chemotion-repository/api/v1/gate/receiving

The following example show the structure of the data.json file:


```
{
  "samples": {
    "a1c3c816-02d9-4222-940c-8773e716b994": {
      "name": "PH-856-A",
      "molfile": "\n  Ketcher 04172019412D 1   1.00000     0.00000     0\n\n  1  0
0     0  0            999 V2000\n   16.8250   -2.8500    0.0000 O   0  0  0  0  0
0  0  0  0  0  0  0\nM  END\n$$$$\n\n",
      "uuid": "a1c3c816-02d9-4222-940c-8773e716b994"
    }
  },
  "analyses": {
    "a1c3c816-02d9-4222-940c-8773e716b994": [
      {
```

```
        "name": "1H",
        "description": "",
        "extended_metadata": {
          "kind": "CHMO:0000593 | 1H nuclear magnetic resonance spectroscopy (1H
NMR)",
          "index": "0",
          "status": "Confirmed",
          "content": "{\"ops\":[{}]}"
        },
        "uuid": "bdded60c-1f39-4f69-9e77-92c76f9a2065",
        "datasets": [
          {
            "name": "PH-856-A 1H",
            "description": "",
            "extended_metadata": {
              "instrument": "Bruker Ascend 400"
            },
            "attachments": [
              {
                "filename": "PH-856-A 1H.dx",
                "identifier": ":",
                "checksum":
"03d6f1f5e7e01adb2e23c392aa9a7600a5e23d5e313ecb56dc2cae414bb99a4d",
                "content_type": ""
              }
            ]
          }
        ]
      }
    ]
  }
}
```


Attachment files can also be added by adding more parts to the mulipart request and with having the uuid attachment identifier as key. (For an example with the previous data.json, one will add **-F f49f252c-dd7f-4e0c-9fef-a6f043d0d431=@…** to the curl request options.)

7. Information on entities that occur in more than one version

The repository’s processes register different versions of the same entity “molecule”. Entries such as samples are considered to belong to the same parent molecule, if the InChIKey is the same. New submissions referring to the same parent are considered as new versions and the new samples including their analytical data are referenced with DOIs that contain a numeric version indicator. The version indicator is separated from the InChIKey descriptor in the DOI name by a dot.

Example:

Parent DOI (sample is version 1 of the molecule):
https://dx.doi.org/10.14272/FWCFZCXASGUOMH-UHFFFAOYSA-N.1

A new submission will lead to the sample DOI:
https://dx.doi.org/10.14272/FWCFZCXASGUOMH-UHFFFAOYSA-N.2

8. Further details: IUPAC name versus formula

The IUPAC name of molecules is retrieved from PubChem. For molecules which are known to PubChem, the name is directly given with the publication in the repository. For new molecules, the name is updated automatically after successful registration of the submission to PubChem. If the update from PubChem is pending or in case of invalid data matching with PubChem, the formula of the molecule is given.

[1] https://github.com/rails/rails, last accessed: 04/17/2020
[2] https://github.com/facebook/react/, last accessed: 04/17/2020
[3] https://doi.org/10.5281/zenodo.3755769, deposit: 04/17/2020; last accessed: 04/17/2020
[4] Zenodo Source: https://doi.org/10.5281/zenodo.3755645
[5] DOI https://doi.org/10.1186/s13321-018-0277-8
[6] http://doi.org/10.5438/0014
[7] http://www.openarchives.org/
[8] https://pubchem.ncbi.nlm.nih.gov/upload/, last accessed: 04/17/2020
[9] S. Kim, P. A. Thiessen, E. E. Bolton, J. Chen, G. Fu, A. Gindulyte, L. Han, J. He, S. He, B. A. Shoemaker, J. Wang, B. Yu, J. Zhang, S. H. Bryant, *Nucleic Acids Res.* **2016**, *44*, D1202–D1213. https://doi.org/10.1093/nar/gkv951
